# E-Cigarettes and Smoking Cessation: Evidence from a Systematic Review and Meta-Analysis

**DOI:** 10.1371/journal.pone.0122544

**Published:** 2015-03-30

**Authors:** Muhammad Aziz Rahman, Nicholas Hann, Andrew Wilson, George Mnatzaganian, Linda Worrall-Carter

**Affiliations:** 1 The Mary MacKillop Institute for Health Research, Australian Catholic University, Melbourne, Australia; 2 St Vincent’s Centre for Nursing Research (SVCNR), Australian Catholic University, Melbourne, Australia; 3 The Cardiovascular Research Centre (CvRC), Australian Catholic University, Melbourne, Australia; 4 The University of Melbourne, Melbourne, Australia; 5 St Vincent’s Hospital, Melbourne, Australia; 6 School of Allied Health, Faculty of Health Sciences, Australian Catholic University, Melbourne, Australia; Centre for Addiction and Mental Health, CANADA

## Abstract

**Background:**

E-cigarettes are currently being debated regarding their possible role in smoking cessation and as they are becoming increasingly popular, the research to date requires investigation.

**Objectives:**

To investigate whether the use of e-cigarettes is associated with smoking cessation or reduction, and whether there is any difference in efficacy of e-cigarettes with and without nicotine on smoking cessation.

**Data Sources:**

A systematic review of articles with no limit on publication date was conducted by searching PubMed, Web of Knowledge and Scopus databases.

**Methods:**

Published studies, those reported smoking abstinence or reduction in cigarette consumption after the use of e-cigarettes, were included. Studies were systematically reviewed, and meta-analyses were conducted using Mantel-Haenszel fixed-effect and random-effects models. Degree of heterogeneity among studies and quality of the selected studies were evaluated.

**Results:**

Six studies were included involving 7,551 participants. Meta-analyses included 1,242 participants who had complete data on smoking cessation. Nicotine filled e-cigarettes were more effective for cessation than those without nicotine (pooled Risk Ratio 2.29, 95%CI 1.05-4.97). Amongst 1,242 smokers, 224 (18%) reported smoking cessation after using nicotine-enriched e-cigarettes for a minimum period of six months. Use of such e-cigarettes was positively associated with smoking cessation with a pooled Effect Size of 0.20 (95%CI 0.11-0.28). Use of e-cigarettes was also associated with a reduction in the number of cigarettes used.

**Limitations:**

Included studies were heterogeneous, due to different study designs and gender variation. Whilst we were able to comment on the efficacy of nicotine vs. non-nicotine e-cigarettes for smoking cessation, we were unable to comment on the efficacy of e-cigarettes vs. other interventions for cessation, given the lack of comparator groups in the studies included in this meta-analysis.

**Conclusions:**

Use of e-cigarettes is associated with smoking cessation and reduction. More randomised controlled trials are needed to assess effectiveness against other cessation methods.

## Introduction

Smoking accounts for more deaths and diseases worldwide than any other modifiable risk factors.[1, 2] Literature suggests that approximately three quarters of smokers want to quit; however, smoking is highly addictive and smoking cessation is difficult with frequent relapses common amongst those who try to quit.[1] There is ongoing research on the effectiveness of various smoking cessation interventions. Nicotine replacement therapy (NRT), bupropion, varenicline and cytisine medications have been shown to improve the likelihood of quitting, with varenicline showing the greatest benefit. However, these products have relatively low consumer appeal and product satisfaction.[3, 4] Evidence suggests that psychosocial smoking cessation interventions such as behavioural counselling, telephone support and self-help interventions are effective; behavioural interventions combined with pharmacotherapy increase the success rate of quitting.[5, 6]

Over recent years, electronic cigarettes (e-cigarettes) have gained the attention of smokers due to their ability to closely simulate the aesthetic and behavioural experience of smoking, as well as delivering a dose of nicotine without involving the combustion of tobacco. E-cigarettes may, therefore, have potential roles in both smoking cessation and tobacco harm reduction.[7, 8] In our recent literature review on the e-cigarette phenomenon, we found there was a need for further research to answer key questions about the safety, patterns of use, effectiveness for smoking cessation and regulatory issues associated with the use of e-cigarettes.[9]

Although the vast majority of e-cigarette users are current smokers, they appear to fall into two distinct groups—those using them to quit smoking and those using them recreationally with no intention of quitting.[9, 10] The presence of recreational e-cigarettes users has raised concerns whether e-cigarettes act as genuine smoking cessation aids or merely ‘bridging’ products, which perpetuate smokers’ nicotine addictions by enabling them to smoke in environments where smoking has been banned, or ‘gateway’ products which hook first time users into nicotine.[10, 11] These concerns have arisen from the studies on initial use of e-cigarettes, however, it is also feasible that entrenched smokers may be able to reduce their tobacco use by substituting e-cigarettes for cigarettes. In terms of the safety of e-cigarettes, while there have been no data on their long term health effects, a substantial body of research now exists reporting mixed findings about the toxicity of their refill solutions, with the variation apparently due to divergent testing methods.[12–14] Similarly, there is conflicting evidence as to whether e-cigarettes are effective for smoking cessation and this creates a pressing dilemma for regulatory authorities which seek to minimise harms without stifling a potentially beneficial product.[10, 11]

Therefore, clarity is required on two key issues; whether they are safe, and whether they are effective aids for smoking cessation. If scientific evidence demonstrates that e-cigarettes are safe and effective for smoking cessation, they are likely to become additional tools for smoking cessation programs and tobacco harm reduction strategies. On the other hand, if they are shown to be unsafe or ineffective for smoking cessation, regulatory decisions which limit their sales and use may be expedited to minimise their use as bridging products which perpetuate or initiate nicotine addiction. Quality evidence to inform these decisions is scarce and a comprehensive systematic review and meta-analysis on this issue has not been undertaken so far. Therefore, the objective of this paper was to conduct a systematic review and meta-analyses to investigate whether long-term use of e-cigarettes among current smokers was associated with smoking cessation or reduction, and whether there is any difference in efficacy of e-cigarettes with and without nicotine on smoking cessation.

## Methods

### Study selection

The team agreed on the search terms, scope and approach for this systematic review and meta-analyses. Two researchers conducted a comprehensive literature search according to the method recommended by the Cochrane Collaboration. Selected studies were evaluated using the Preferred Reporting Items for Systematic Reviews and Meta-analyses (PRISMA) guidelines.[15, 16] In line with the objective of this systematic review, use of e-cigarettes was selected as the main exposure variable and smoking cessation as the predominant outcome variable. We reviewed published studies including randomised controlled trials (RCTs), cohort, case-control and cross-sectional studies, if they assessed the efficacy of e-cigarettes in achieving smoking abstinence or reduction in cigarette consumption, among current smokers who had used the devices for six months or more. Studies with additional outcomes pertaining to smoking reduction were permitted, while those with primary endpoints concerning other issues such as attenuation of withdrawal symptoms were excluded.

### Search strategy

**Fig 1:**
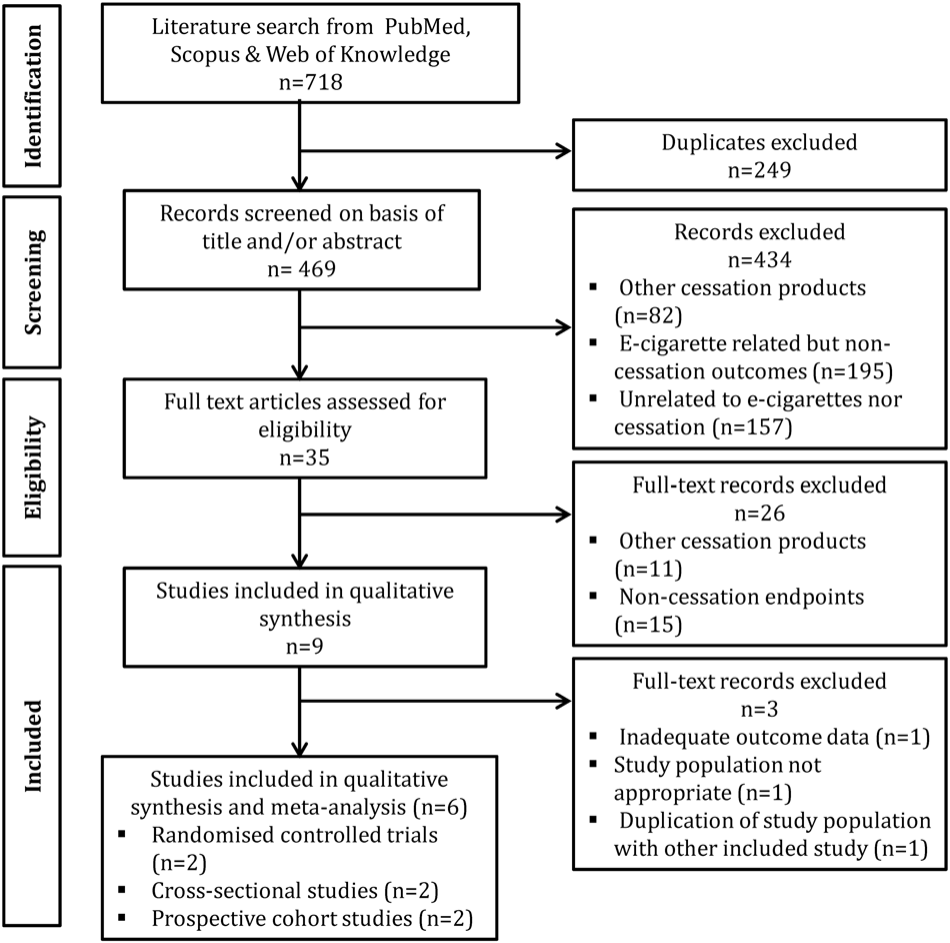
Search strategy for identification and selection of studies investigating the efficacy of electronic cigarettes for smoking cessation and reduction (PRISMA flowchart).

Fig 1 depicts the search strategy used and number of records identified and excluded at each step. PubMed, Web of Knowledge and Scopus databases were searched using the following terms: “electronic cigarettes OR e-cigarettes” AND “smoking cessation OR quit smoking”. Further search criteria were studies published in English and conducted on humans. There was no limit on publication date. The databases were last searched in May, 2014. The combined search revealed 718 articles, from which 249 were removed as duplicates. Titles and abstracts were reviewed for the remaining articles, from which 434 were excluded and 35 full-text articles were reviewed against the inclusion criteria.

### Study selection and data extraction

Nine studies were initially included, but three were subsequently excluded because they investigated smoking cessation in a schizophrenic group of patients.[17–19] These exclusions were made on the basis of inadequate outcome data and/or study populations that were either incongruent or overlapping with the other included studies.[20–25]

### Data analyses

Meta-analyses were performed using the metan Stata statistical program (version 13, StataCorp, College Station, TX), and were conducted with two objectives: 1) to compare the effect of e-cigarettes with and without nicotine on abstinence from tobacco smoking in order to evaluate the device’s associated placebo effect, and 2) to evaluate the long-term association between the use of e-cigarettes (i.e., after at least six months use) and smoking cessation. This was investigated by calculating a pooled proportion of quitters as reported in the meta-analysed studies. Subjects lost to follow up were counted as smokers (consistent with intention-to-treat methods). In addition to these objectives, the quality of included studies was also assessed.

Findings from the two included RCTs by Bullen et al. and Caponnetto et al. were meta-analysed to answer the study’s first objective.[20, 21] Meta-analyses were performed using a Mantel-Haenszel fixed effects model.[26] The pooled risk ratio (RR) with 95% confidence interval (CI) was calculated. The RR was defined as the ratio of risk of abstinence from smoking among those exposed to nicotine enriched e-cigarettes and the risk of abstinence among those using non-nicotine enriched e-cigarettes. If the value of 1 was not within the 95% CI, the RR was statistically significant at the 5% level (p<0.05). The I^2^ statistic was calculated to demonstrate the degree of heterogeneity—that is, the percentage of variation across studies that is not due to chance.[27]

For the second objective, an overall proportion of abstinence from the set of proportions reported in each of the six included studies was calculated using a random-effects meta-analysis model using DerSimonian and Laird method.[28] This method incorporates an estimate of the between-study variation into both the study weights and the standard error of the estimate of the common effect. The precision of an estimate from each included study was represented by the inverse of the variance of the outcome pooled across all participants.[29] Less precise estimates have larger variances, so the inverse of variance is smaller for studies with less precise estimates. The fixed effects model was utilised when running the sub analyses by study designs. The pooled effect size (ES) (estimated by the pooled proportion) with 95% CI was calculated. If the value of the pooled proportion ‘zero’ was not within the 95% CI, the ES was statistically significant at the 5% level (P<0.05).

### Quality assessment

The quality of the studies included in the meta-analyses was assessed at the entire study level as well as at the outcome level, in the manner recommended by the Cochrane Collaboration using the Downs and Black instrument.[15, 30] For RCTs, the checklist produced by van Tulder et al. as part of the Cochrane Back Review Group was used, while the checklist provided by Downs and Black was used to assess bias in the observational studies.[30, 31] These tools were simultaneously used to make an assessment of the risk of bias affecting the findings of the studies. We also examined whether all outcomes were reported and reasons behind any exclusion. The quality of the studies was independently assessed by three researchers. The degree of agreement between researchers was calculated together with Cohen’s Kappa coefficient to measure inter-rater agreement.

## Results

### Characteristics of included studies

Table 1 presents summary data and key findings for all six included studies.

**Table 1:** Summary of studies reporting smoking cessation after the use of e-cigarettes.

Source	Type of study	Study population	Sample Size	Intervention	Comparator	Length of follow-up	Quit Intention	Key findings	Comments
Brown et al. (2014)	Cross-sectional study	Members of public responding to national survey	5863	Survey of current smokers using an e-cigarette to quit	NRT	12 months	Intending to quit	1) Smoking abstinence reported more commonly by e-cigarette users than users of over-the-counter NRT (OR 2.23) and those quitting smoking unaided (OR 1.38)	Sample adequately representative of adult population in England.
								2) Adjusted odds of non-smoking among e-cigarettes were 1.63 times higher than users of NRT and 1.61 times higher than those using no aid	Subjects recruited over long period of time.
									Adequate allowance made for confounders in a large population.
Bullen et al. (2013)	Randomised Controlled Trial	Members of public responding to newspaper ads	657	12-weeks' e-cigarette use	Placebo e-cigarettes	6 months	Intending to quit	1) Complete abstinence rates of 7.3% (nicotine e-cigarettes), 4.1% (placebo e-cigarettes) and 5.8% (patches)	Underpowered to conclude superiority of e-cigarettes compared to NRT for smoking cessation.
								2) ≥50% cig/day reduction in 57% nicotine e-cigarettes group, 45% placebo e-cigarettes group and 41% patches group	
Caponnetto et al. (2013)	Randomised Controlled Trial	Members of public responding to newspaper ads	300	12-weeks' e-cigarette use	Placebo e-cigarettes	9 months	Not intending to quit	1) Complete abstinence rate of 11% in nicotine e-cigarette groups and 4% in placebo e-cigarette group	Recruitment limited to those not intending to quit.
								2) ≥50% cig/day reduction in 14.5% of nicotine e-cigarette groups combined and 12% of placebo e-cigarettes group	Self-reporting in study diaries, potential source of measurement bias.
									Substantial loss to follow up.
Etter et al. (2014)	Prospective Cohort	Members of public using e-cigarette and smoking cessation websites	477	Survey of dual e-cigarette and tobacco users over time	None	12 months	Mixed intentions	1) Complete self-reported cessation rate at 1 month of 22% among current smokers (n = 50)	Potential for selection bias as participants drawn from website users.
								2) Complete self-reported cessation rate at 12 months of 46% among current smokers (n = 35)	Smoking abstinence and reduction were not biochemically verified.
								3) Reduction in cig/day of 10.5 among current smokers at 12 months	
Polosa et al. (2013)	Prospective Cohort	Staff from one hospital	40	24-weeks' e-cigarette use	None	24 Months	Not intending to quit	1) Complete abstinence rate of 12.5%	Selected group of subjects.
								2) Sustained ≥50% cig/day reduction in 27.5% subjects	Small sample size.
									Substantial loss to follow up.
Siegel et al. (2011)	Cross-sectional study	E-cigarette users of one brand responding to email	216	Survey of dual e-cigarette and tobacco users over time	None	6 months	Mixed intentions	1) Complete abstinence rate of 31%	Potential for significant selection bias, as participants were self-selected by responding to email sent to buyers of one e-cig brand.
								2) Any reduction in cig/day in 66.8% subjects	Smoking abstinence and reduction were not biochemically verified.

### Study type

Of the six included studies, two were RCTs, two were cross-sectional studies, and two were prospective cohort studies.[20–25]

### Participants

The combined sample size from the selected studies was 7,551. Recruitment methods were similar across most studies, and used either newspaper advertisements, emails to product users or national health agency-run surveys to recruit members of the public.[20–25] Two studies conducted comprehensive randomisation processes of participants.[20, 21] Inclusion and exclusion criteria were also largely common to all selected studies. The predominant inclusion criteria were adult current smokers (although exact definition of smoking status varied), while the main exclusion criteria were comorbid cardiovascular disease, diabetes, major depression and other psychiatric disorders.

### Smoking status and quit intentions

All participants in the included studies were current smokers.[20–25] In two of the studies participants were not intending to quit smoking prior to use of an e-cigarette,[21, 23] while remaining studies’ participants were either intending to quit or had mixed intentions.[20, 22, 24, 25]There was slight variation between the studies in the way smoking status was defined. Most studies defined current smokers as those who had smoked at least 10 cigarettes per day for a period of years.[20, 21, 23] Two studies did not explicitly define current smoking status, however, participants self-reported themselves as either daily or occasional smokers.[22, 25] Finally, Siegel et al. defined it as participants having smoked ≥100 cigarettes in their lifetime.[24] Recruitment criteria regarding participants’ intention to quit varied across studies; two studies recruited those who were intending to quit, two not intending to quit, and the remaining two studies’ participants had mixed intentions.

### Intervention

The intervention implemented in three of the six studies was ad lib use of e-cigarettes, which were provided to participants for the duration of the study period.[20, 21, 23] The remaining studies were cross-sectional and cohort in design, and did not include an intervention, but similarly investigated participants’ ad lib use of e-cigarettes over the study period.[22, 24, 25]

### Comparator

Two of the selected studies (both RCTs) included formal comparator groups or interventions.[20, 21] One used groups assigned to nicotine patches and placebo e-cigarettes as comparators,[20] while the other just used a group assigned to placebo e-cigarettes as a comparator.[21] One of the cross-sectional studies compared smoking cessation rates among e-cigarette users to those using NRT and those quitting unaided.[25]

### Outcomes

The primary outcome for five of the studies was either abstinence from smoking at the end of the designated study period or reduction in smoking (as measured by number of cigarettes per day).[20, 21, 23–25] The remaining study primarily investigated longitudinal usage patterns of e-cigarettes but measured smoking cessation and reduction as a secondary outcome.[22] Adverse events and withdrawal symptoms were specifically assessed in both RCTs [20, 21] and in one cohort study.[24]

### Definition of smoking cessation

Studies defined smoking cessation in one of two ways. Three studies defined it as complete self-reported abstinence from tobacco cigarettes over a given portion of the follow-up period, verified by an exhaled carbon monoxide (eCO) measurement of either ≤7ppm or ≤10ppm.[20, 21, 23] The remaining studies did not explicitly define smoking cessation; but similar to the others, implied complete self-reported abstinence from tobacco without the requirement for biochemical verification.[22, 24, 25]

### Efficacy of e-cigarettes with and without nicotine on smoking cessation

**Fig 2:**
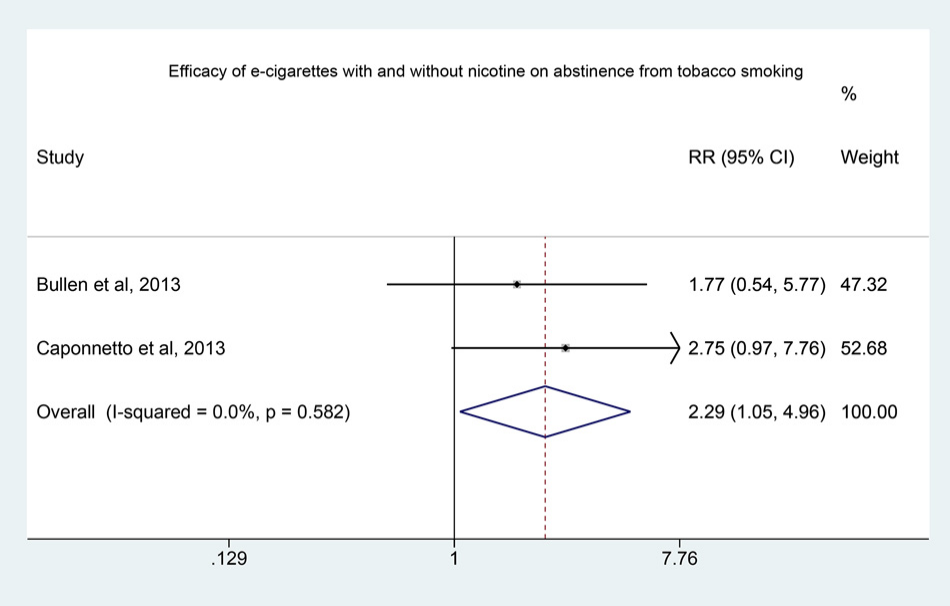
Forest Plot of the effectiveness of e-cigarettes with and without nicotine in smoking cessation.

Number of those who stopped and did not stop smoking among users of nicotine and non-nicotine enriched e-cigarettes is shown in Table 2. E-cigarettes with nicotine were more effective for smoking cessation than e-cigarettes without nicotine. Pooled data from the two included RCTs showed a statistically significant benefit of nicotine filled e-cigarettes compared to those without nicotine (pooled Risk Ratio 2.29, 95% CI 1.05 to 4.97). Pooled RR is shown in Fig 2, where the location of the diamond represents the estimated effect size, being statistically significant as it is larger than 1, and the width represents its precision also depicted by the confidence interval. None of the RCTs was heterogeneous (I^2^ = 0.0%, P = 0.6). Based on these findings, only e-cigarettes with nicotine were considered in the subsequent analyses.

**Table 2:** Characteristics of the two randomised controlled trails included in the meta-analysis that assessed the efficacy of e-cigarettes with and without nicotine for smoking cessation.

Authors, year, study design	e-cigarettes with nicotine		e-cigarettes without nicotine		RR (95% CIs)	SE (log RR)	M-H weight
	Stopped smoking (n)	Continued smoking (n)	Stopped smoking (n)	Continued smoking (n)			
Bullen et al, 2013, RCT	21	268	3	70	1.77 (0.5–5.8)	0.6	47.3
Caponnetto et al, 2013, RCT	22	178	4	96	2.75 (0.9–7.8)	0.53	52.7

RCT: randomized controlled trial, RR: relative risk, CIs: confidence intervals, SE: standard error, M-H: Mantel–Haenszel

### Association between the use of e-cigarettes and smoking cessation

Subjects with complete information on smoking cessation (i.e., those who had information on smoking cessation at the end of at least six months of follow up) were eligible to be included in the meta-analysis. Of the original samples (n = 3,865 men and n = 3,686 women) reported in the six studies, a total sample of 1,242 was meta-analysed. (Table 3)The latter had a weighted average age of 41.5 (SD 13.9) years with a male to female proportion of 1.4, and an average daily cigarette consumption of 16.2 cigarettes per day. Of the 1,242 smokers who reported using nicotine enriched e-cigarettes, 224 (18%) reported complete smoking cessation after a minimum 6-month use of e-cigarettes.

**Table 3:** Proportion of smoking quitters in nicotine enriched e-cigarette users by study.

Authors, year	Study design	Total sample (N)	Stopped smoking (n)	Proportion (95% CIs)	SE	Weight^#^
Brown et al, 2014	Cross sectional	464	93	0.20 (0.16, 0.24)	0.02	18.8
Bullen et al, 2013	RCT	289	21	0.07 (0.04, 0.10)	0.02	19
Caponnetto et al, 2013	RCT	200	22	0.11 (0.07, 0.15)	0.02	18.5
Etter et al, 2013	Cohort	35	16	0.46 (0.29, 0.62)	0.08	11
Polosa et al, 2013	Cohort	40	5	0.12 (0.02, 0.23)	0.05	15
Siegel et al, 2011	Cross sectional	214	67	0.31 (0.25, 0.38)	0.03	17.6

RCT: randomized controlled trial, CIs: confidence intervals, SE: standard error

^#^ Weight: the weight was based on a random effects model using DerSimonian and Laird method

**Fig 3:**
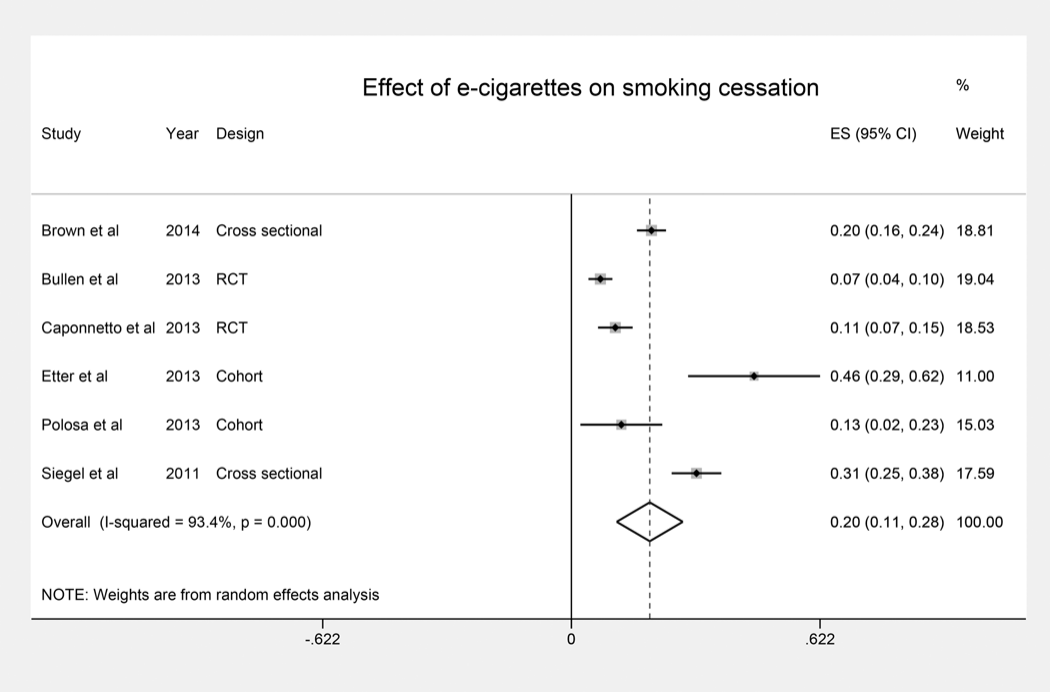
Forest Plot of the association between nicotine-enriched e-cigarettes and smoking cessation.

Pooled data from two RCTs, two cohort and two cross-sectional studies on the association of nicotine enriched e-cigarettes with smoking cessation revealed a statistically significant effect with an overall pooled Effect Size of 0.20 (95% CI 0.11 to 0.28) as shown in the forest plot (Fig 3). As expected, there was considerable heterogeneity among the studies (I^2^ = 93.4%, p<0.001). A meta-regression model showed that 98% of this heterogeneity was caused by the research study design, and the variation in the proportion of males to females in each of the included studies. Running the analyses separately by study design showed similar significant findings (Table 4). However, the pooled effect size derived from the RCTs was smaller than those observed in observational studies. Available data did not allow us to conduct the analyses by gender. A further stratified analysis was conducted by smoking abstinence verification method. The two RCTs and one cohort study verified cessation using biochemical methods.[20–22] The pooled ES of these three studies was 0.09 (0.06, 0.11). These three studies were not heterogeneous, I2 = 20%, P = 0.3. In the remaining studies, abstinence was self-reported with a pooled ES of 0.24 (0.21, 0.27). Studies with self-reported data were heterogeneous (I^2^ = 88%, p<0.001).

**Table 4:** Pooled proportion of smoking quitters among nicotine enriched e-cigarettes by study design: fixed effects meta-analysis model.

Study design	Pooled ES (95% CIs)	I-squared (variation in ES attributable to heterogeneity)	Heterogeneity chi-squared
**RCTs**	0.08 (0.06, 0.11)	48.20%	p = 0.165
Bullen et al, 2013			
Caponnetto et al, 2013			
**Cohort studies**	0.22 (0.02, 0.23)	91.10%	p = 0.001
Etter et al, 2013			
Polosa et al, 2013			
**Cross sectional studies**	0.23 (0.20, 0.26)	89.40%	p = 0.002
Brown et al, 2014			
Siegel et al, 2011			

### E-cigarettes for smoking reduction

A qualitative analysis was conducted to determine whether use of e-cigarette reduced cigarette consumption (rather than cessation) among the current smokers. All studies reported substantial rates of smoking reduction among participants (Table 1).[20–25] Three studies reported >50% reduction in daily cigarette consumption.[20, 21, 23]

### Quality assessment

Risk of bias in the included studies was evaluated using specific quality assessment tools and completed by two researchers independently. Potential bias was accounted for in 57% of all items assessed in all studies, not accounted for in 24%, and in the remaining 19% such bias was either not applicable or hard to assess due to missing information. Percent agreement between the assessors was 78%, with a Kappa coefficient 0.62 (95% CI 0.50–0.69) showing substantial agreement.

Two RCTs had limited risk of bias.[20, 21] In the study by Bullen et al. there was a risk of selection bias due to recruitment via community newspapers and being unable to blind participants receiving intervention.[20] The study by Caponnetto et al. was similarly at risk of selection bias due to recruitment via local newspapers. This study also had a loss-to-follow-up of 39%, which is not unusual for other studies of smokers who were unwilling to quit. Furthermore, since those participants were treated as failures according to intention-to-treat analysis, reported cessation rates would, if anything, have been underestimated.[21]

Four observational studies were at risk of selection bias as they either used selected groups as participants or had low response rates to recruitment methods.[22–25] The one possible exception to this is the study by Brown et al. which included participants from a large-scale national sampling survey. Those four studies also used self-reporting as the predominant method to record exposure, making them susceptible to information bias.[22–25] This bias was minimised in the study by Polosa et al., which used validated means (eCO) to verify outcomes objectively.[23]

## Discussion

This systematic review and meta-analysis indicates an association between the use of nicotine-enriched e-cigarettes and smoking cessation. We also report that e-cigarettes containing nicotine are more effective at aiding smoking cessation than e-cigarettes without nicotine, and that e-cigarettes of both types may help ongoing smokers by reducing the number of tobacco cigarettes they use. The association between the use of e-cigarettes and smoking cessation was consistently observed in all included studies, also seen in different study designs, and abstinence verification method. Studies that relied on a more objective biochemical abstinence verification method had a lower pooled effect size than the studies that had self-reported information. Nonetheless, the effect sizes in each verification method remained statistically significant. Our results indicate that nicotine enriched e-cigarettes may prove to be a useful smoking cessation method.

We compared findings from this meta-analysis to cessation rates known to be achievable with existing NRTs. Two studies reported that the 12-month quit rate achieved using NRTs was approximately 10%, and would not exceed this level in the longer term.[3, 4] Our meta-analyses demonstrated a higher smoking cessation rate of 20% achieved with e-cigarettes, suggesting that factors beyond nicotine replacement alone may contribute to smoking cessation.[22] A feasible explanation for this is that e-cigarettes closely mimic the behavioural and aesthetic aspects of smoking, whereby the instinctive physical behaviours and cue responses that smokers are accustomed to are satisfied alongside their pharmacological needs. This concept has been elucidated in a qualitative study, in which users cite multiple non-pharmacological reasons for their satisfaction with e-cigarettes.[32] This includes sensory and behavioural simulation of smoking, as described by Caponnetto et al. in a study using a nicotine-free inhalator to demonstrate the impact of handling and manipulation in promoting smoking cessation.[33] Non-pharmacological drivers for e-cigarette users’ satisfaction included the social benefits of being part of a ‘vaping’ community and counteracting the negative stigma attached to smoking.[32]

Studies investigating patterns of e-cigarette consumption and user beliefs have similarly found non-pharmacological reasons for use and satisfaction.[10, 34, 35] These include financial reasons (as e-cigarettes are cheaper than tobacco), a perception that e-cigarettes are less harmful than tobacco and the ability to use them in places where smoking is banned.[10, 34, 35] Smokers may be attracted to and satisfied with e-cigarettes for these reasons beyond any contribution to smoking cessation. This would explain our finding of comparable cessation rates regardless of intention to quit smoking, and for higher cessation rates than those reported by studies investigating NRTs.[3, 4]

Another key finding of our study is that use of e-cigarettes was associated with a reduction in the number of cigarettes used, which is important in light of the substantial body of evidence demonstrating that gradual reduction in cigarette consumption aids future quit attempts.[36–38] This indicates a potential role for e-cigarettes in tobacco harm reduction programs, in addition to a possible role as an alternative smoking cessation tool. It also suggests that in these cases at least, dual use of e-cigarettes does not necessarily perpetuate or exacerbate smokers’ tobacco addiction and use, as some public health researchers have warned.

In analysing our findings we sought to examine the effect of potential confounders, including study design and smokers’ intention to quit smoking. We found that the positive association between use of nicotine-enriched e-cigarettes and smoking cessation was not affected by study designs. When meta-analyses were calculated according to study designs, the results remained significant within each study type. However, the pooled effect size derived from the RCTs was smaller than that found in either cohort or cross-sectional studies. Cessation rates were lower in all of those three studies, where biochemical abstinence verification methods were used.[20, 21, 23] Smokers’ intention to quit did not appear to affect this positive association. However, we note that, unlike traditional RCTs, these studies did not investigate the effect of a medication on a disease state, but rather investigated the use of a non-medication intervention (e-cigarettes) on a behaviour (smoking).

This review represents the most comprehensive evidence on e-cigarettes currently available, and provides important and timely information for regulatory authorities and policymakers. To date, the Food and Drug Administration (FDA) in the US and parliaments in Europe and the UK have been under pressure to decide how strictly to regulate e-cigarettes, but devoid of availability of substantial evidence regarding their cessation-related benefits versus safety or addiction-related drawbacks.[39–41] Our findings indicate that e-cigarettes may be an effective alternate smoking cessation tool will provide timely evidence to help informing this important regulatory debate. Our findings may also provide important updates to other stakeholders, including the wider research community and healthcare providers. Importantly, we note that the long term health effects of e-cigarettes use are yet to be examined.

Limitations of this review include the paucity of available studies, biases within existing studies, and their heterogeneity and variable quality. Studies were variously affected by small sample sizes and methodological variation in data collection, rendering them susceptible to varying degrees of selection and information bias. Furthermore, whilst we were able to comment on the efficacy of nicotine e-cigarettes vs. non-nicotine e-cigarettes for smoking cessation, we were unable to comment on the efficacy of e-cigarettes vs. other interventions for cessation, given the lack of comparator groups in the studies included in this meta-analysis. Similarly, since the pooled effect size derived from the RCTs was considerably smaller than those observed in observational studies, the overall effect size from all studies combined may have been overestimated.

This review highlights the need for further research on e-cigarettes. The available data did not allow us to conduct sub analysis by gender which is an important factor. It is important to explore gender-related heterogeneity between studies, so it can be determined whether the overall trend differs according to this factor. Large-scale, randomised trials are required to validate our study findings, and re-confirm the association between e-cigarette use and smoking cessation. It is imperative that the safety and contents of e-cigarettes also need to be investigated and confirmed if they are to be used as smoking cessation aids or in tobacco harm reduction programs. Finally, a wider cost-effectiveness analysis of the devices, weighing up the various cessation and harm reduction benefits against any safety or other concerns would be highly valuable from a public health perspective. If the safety of e-cigarettes is proven, e-cigarettes may assist healthcare providers to address smoking cessation challenges more effectively.

## Conclusions

This systematic review and meta-analyses assessed the findings of six studies which reported smoking cessation after using e-cigarettes. We found an association between nicotine-enriched e-cigarette use and smoking cessation, suggesting that the devices may be an effective alternative smoking cessation method. We also found that use of e-cigarettes was also associated with a reduction in the number of cigarettes used, suggesting they may also have a role in tobacco harm reduction programs. To our knowledge, this is the most comprehensive evidence to date on this issue, and while there are a number of important implications for further research, these findings provide timely information to inform regulatory strategies.

## Supporting Information

S1 PRISMA Checklist PRISMA Checklist. (PDF)
